# Genomic Biomarkers to Predict Resistance to Hypomethylating Agents in Patients With Myelodysplastic Syndromes Using Artificial Intelligence

**DOI:** 10.1200/PO.19.00119

**Published:** 2019-09-20

**Authors:** Aziz Nazha, Mikkael A. Sekeres, Rafael Bejar, Michael J. Rauh, Megan Othus, Rami S. Komrokji, John Barnard, Cameron B. Hilton, Cassandra M. Kerr, David P. Steensma, Amy DeZern, Gail Roboz, Guillermo Garcia-Manero, Harry Erba, Benjamin L. Ebert, Jaroslaw P. Maciejewski

**Affiliations:** ^1^ Cleveland Clinic, Cleveland, OH; ^2^ University of California San Diego, San Diego, CA; ^3^ Queen’s University, Kingston, Ontario, Canada; ^4^ Fred Hutchinson Cancer Research Center, Seattle, WA; ^5^ Moffitt Cancer Center, Tampa, FL; ^6^ Dana-Farber Cancer Institute, Harvard Medical School, Boston, MA; ^7^ The Johns Hopkins University School of Medicine, Baltimore, MD; ^8^ Weill Cornell University, New York, NY; ^9^ MD Anderson Cancer Center, Houston, TX; ^10^ Duke University, Durham, NC; ^11^ Brigham and Women’s Hospital, Harvard Medical School, Boston, MA

## Abstract

**PURPOSE:**

We developed an unbiased framework to study the association of several mutations in predicting resistance to hypomethylating agents (HMAs) in patients with myelodysplastic syndromes (MDS), analogous to consumer and commercial recommender systems in which customers who bought products A and B are likely to buy C: patients who have a mutation in gene A and gene B are likely to respond or not respond to HMAs.

**METHODS:**

We screened a cohort of 433 patients with MDS who received HMAs for the presence of common myeloid mutations in 29 genes that were obtained before the patients started therapy. The association between mutations and response was evaluated by the Apriori market basket analysis algorithm. Rules with the highest confidence (confidence that the association exists) and the highest lift (strength of the association) were chosen. We validated our biomarkers in samples from patients enrolled in the S1117 trial.

**RESULTS:**

Among 433 patients, 193 (45%) received azacitidine, 176 (40%) received decitabine, and 64 (15%) received HMA alone or in combination. The median age was 70 years (range, 31 to 100 years), and 28% were female. The median number of mutations per sample was three (range, zero to nine), and 176 patients (41%) had three or more mutations per sample. Association rules identified several genomic combinations as being highly associated with no response. These molecular signatures were present in 30% of patients with three or more mutations/sample with an accuracy rate of 87% in the training cohort and 93% in the validation cohort.

**CONCLUSION:**

Genomic biomarkers can identify, with high accuracy, approximately one third of patients with MDS who will not respond to HMAs. This study highlights the importance of machine learning technologies such as the recommender system algorithm in translating genomic data into useful clinical tools.

## INTRODUCTION

The hypomethylating agents azacitidine (AZA) and decitabine (DAC) have been approved by the Food and Drug Administration and the European Medicine Agency for patients with myelodysplastic syndromes (MDS).^1-4^ Although treatment with these agents is well tolerated, only 30% to 40% of patients will respond to therapy, with the majority achieving hematologic improvement in blood counts and only a minority (10% to 15%) achieving a complete response, the response criterion most reliably associated with improvement in overall survival (OS).^1-4^ More importantly, it may take up to six cycles of treatment for patients to achieve their best response.^5^ Given the limited number of patients who benefit from these agents and the long administration of their treatment, identifying biomarkers that can predict resistance is essential, because it can prevent prolonged exposure to ineffective therapy and unnecessary toxicities and treatment costs can be avoided.

Because clinical variables and patient characteristics have not consistently predicted response to hypomethylating agents (HMAs), molecular data represent a biologic opportunity^6-8^ to enhance patient response rates and outcomes. Although recurrent somatic mutations have been described in several genes in MDS and have implications for disease biology and OS,^9^ the impact of these mutations on response to HMAs remains controversial, with studies evaluating the impact including a small number of patients or a small number of gene sets.^10-14^ For example, some studies have shown that *TP53* mutations may predict a higher response of limited duration to HMAs, whereas others have shown no impact of *TP53* mutations on response.^8,13,14^ Similarly, studies have shown that mutations along methylation pathways, such as *TET2,* may predict higher responses to HMAs, but only in patients with a variant allelic frequency of 10% or more,^11^ whereas a combination of genes such as *ASXL1* mutations with wild-type *TET2* may predict resistance to HMAs.^11^ These approaches do not take into account the genomic heterogeneity or hierarchy of MDS or the association of these mutations with each other. Because identifying a single gene or comutated genes is unlikely to yield an understanding of how these mutations define disease biology or phenotype, an unbiased approach is needed to study the relationship of these abnormalities to each other and to MDS biology.

In this study, we used unbiased, machine learning approaches (a recommender system similar to that used by Netflix or Amazon.com) to assess the impact of molecular data on resistance to HMAs in a large cohort of patients treated with HMAs at different academic institutions, and we validated our results in a population treated in a contemporary prospective clinical trial of HMA therapy^15^ of AZA alone and in combination.

## METHODS

### Patients

For the training cohort, patients treated with either AZA- or DAC-based regimens were included in this study: 230 patients were treated at Cleveland Clinic (between 2002 and 2012); 203 were from other academic institutions (Dana-Farber Cancer Institute [2003 to 2010, n = 42] and MD Anderson Cancer Center [2003 to 2010, n = 103]) or were part of the DACO-020 clinical trial (ADOPT [2005 to 2006, n = 58]).^11^ All patients consented to blood or bone marrow samples at each institution under institutional review board–approved protocols in accordance with each institution policy and the Declaration of Helsinki. More information regarding the patient cohort, response criteria, and validation cohort is included in the Data Supplement.

### DNA Sequencing and Mutational Analysis

A panel of 29 genes that are commonly mutated in MDS and myeloid malignancies was evaluated (Data Supplement). For samples obtained from Cleveland Clinic, genomic DNA was extracted from peripheral blood or bone marrow mononuclear cells before treatment. More information regarding sequencing method is included in the Data Supplement.

### Statistical Analyses

Variables were compared using the Wilcoxon rank sum test and Fisher’s exact test for continuous and categorical variables, respectively. OS was calculated from the date of diagnosis to the date of last follow-up or death (whichever came first), and survival curves were constructed using the Kaplan-Meier method and compared using the log-rank test. Univariate analyses were conducted to evaluate the impact of single mutations on response. A multivariate analysis using logistic regression was conducted and included variables with *P* values of < .1 from univariate analyses. Details regarding the recommender system algorithm are included in the Data Supplement.

## RESULTS

### Patients

A total of 433 patients were included in the final training cohort analysis. The median age at diagnosis was 70 years (range, 31 to 100 years). Table 1 summarizes patient clinical characteristics. Two hundred twenty-eight patients (53%) received AZA (193 [85%] alone and 35 [15%] in combination with other agents), and 205 (47%) received DAC (176 [86%] alone and 29 [14%] in combination with other agents). Cytogenetic analyses per International Prognostic Scoring System (IPSS)–revised (R) criteria^16^ included 234 patients (54%) with very good or good risk, 66 (15%) with intermediate risk, 33 (8%) with poor risk, 66 (15%) with very poor risk, and 34 (8%) not documented (Table 1). A total of 125 (29%) were in the very low or low, 100 (23%) were in the intermediate, 113 (26%) were in the high, and 95 (22%) were in the very high risk category per IPSS-R (Table 1). The 2008 WHO classification for the entire cohort is summarized in Table 1.

**TABLE 1.**
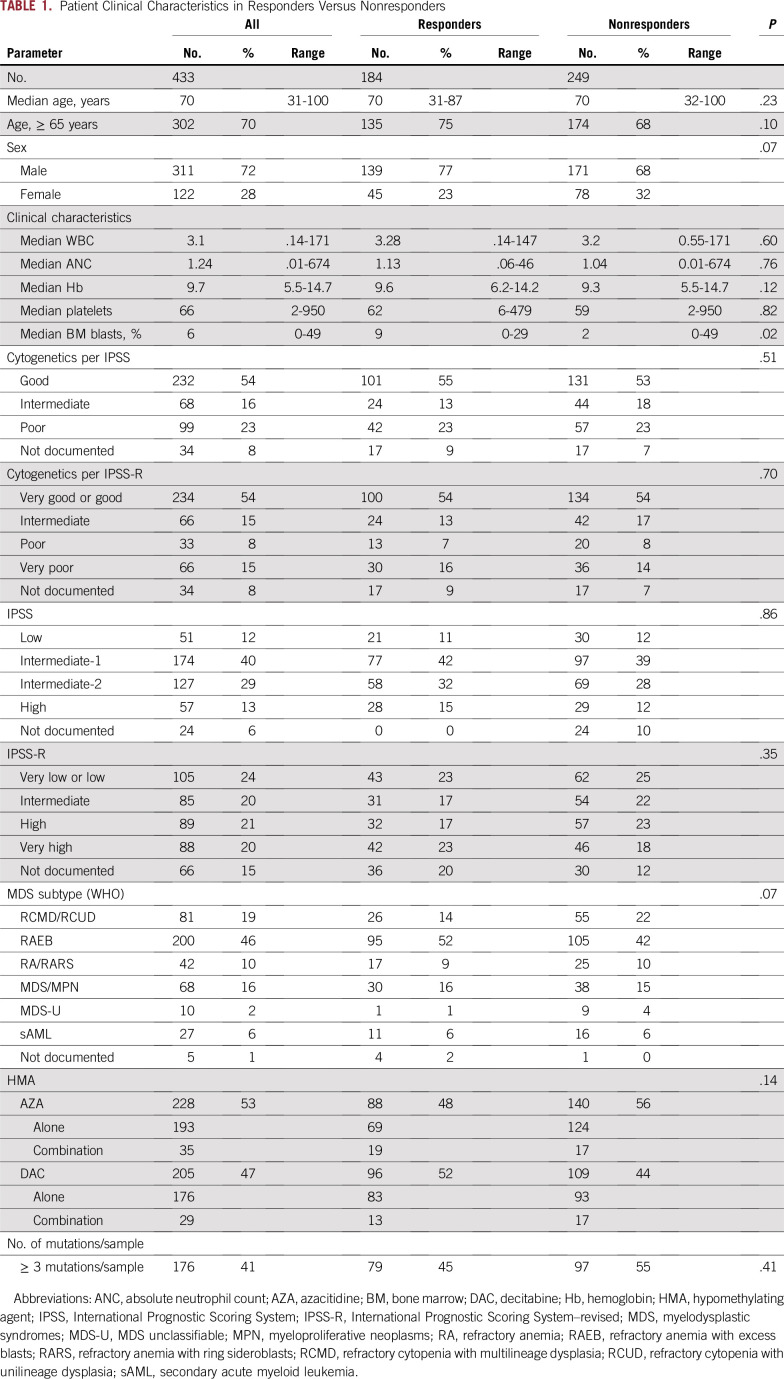
Patient Clinical Characteristics in Responders Versus Nonresponders

### Mutation Distribution

**FIG 1.**
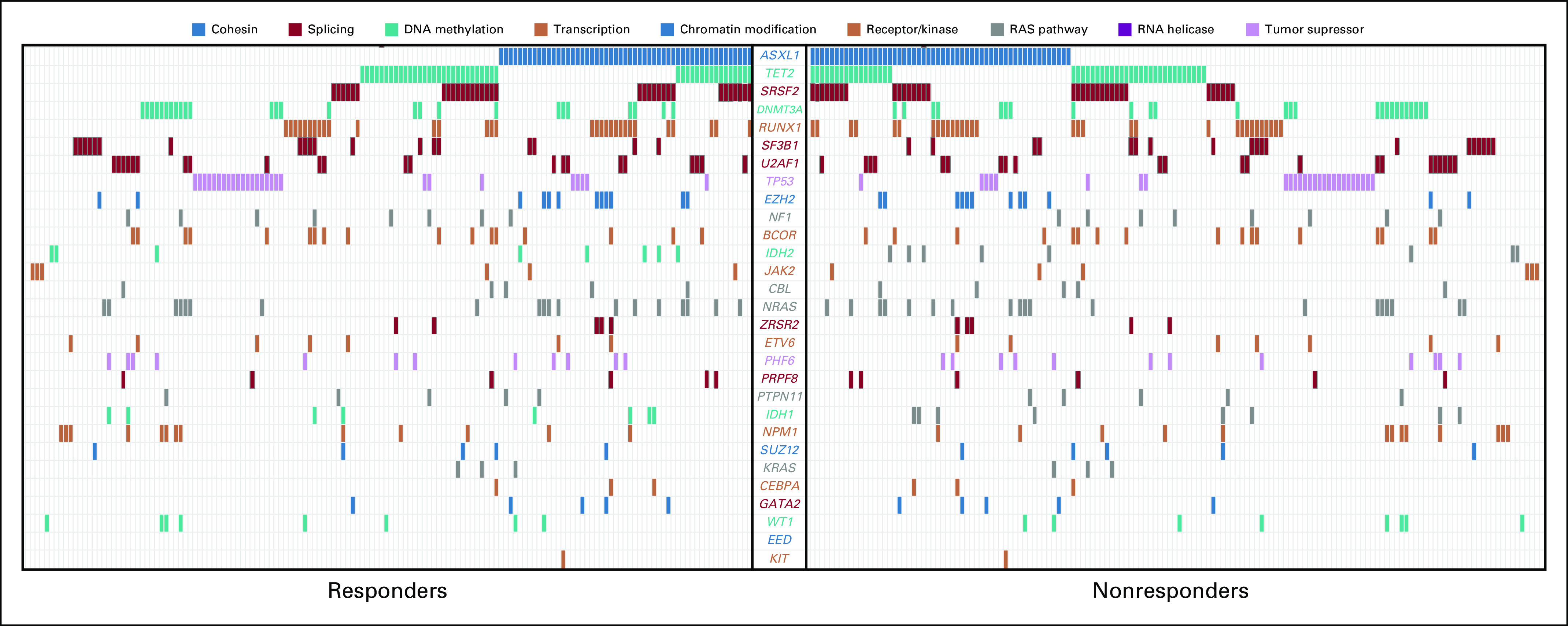
Spectrum of mutations in 433 patients with 29 genes. Each column represents a patient sample and each colored cell represents a mutation of the gene or gene groups listed in the middle. The graph is separated to show the spectrum of mutations in responders compared with nonresponders.

Overall, 367 patients (85%) in the training cohort had a mutation in at least one gene. The median number of mutations per patient was three (range, zero to nine), and a total of 176 patients (41%) had three or more mutations/sample. The most frequently mutated genes were *ASXL1* (31%), *TET2* (22%), *SRSF2* (23%), *RUNX1* (15%), *DNMT3A* (14%), and *SF3B1* (12%; Table 2). The frequency of these mutations in our patient cohort was similar to those identified in other MDS cohorts, with the exception of *ASXL1,* which was slightly higher because it was overrepresented in one cohort^11^(203 patients from other academic institutions). Patterns of mutation association were also similar to those in previous reports (Fig 1). *ASXL1* mutations were commonly commutated with *TET2* in 42 patients (10%), and with *SRSF2* 38 (9%), *RUNX1* 35 (8%), *U2AF1* 24 (6%), and *DNMT3a* 21 (5%; Fig 1).

**TABLE 2.**
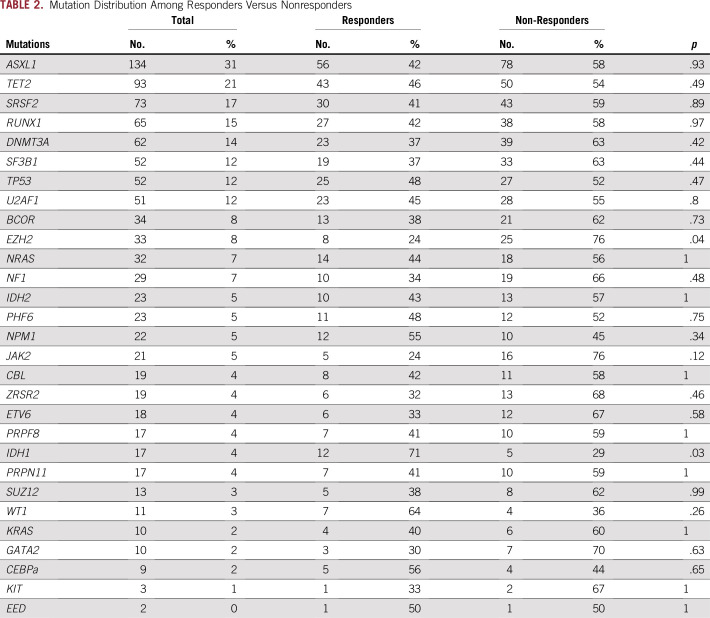
Mutation Distribution Among Responders Versus Nonresponders

### Standard Clinical and Mutational Predictors of Response

The overall response rate to HMAs was 43%, with 109 patients (25%) achieving complete remission (CR), 16 (4%) partial remission (PR), 59 (14%) hematologic improvement (HI), 142 (33%) stable disease, and 107 (24%) progressive disease. In general, clinical characteristics such as age, cytopenias, and treatment regimens did not affect response, with the exception of the median blast percentage in the bone marrow, which was higher among responders compared with nonresponders (9% *v* 2%, *P* = .02; Table 1). Risk stratifications per IPSS and IPSS-R did not affect the overall response rate (Table 1).

No single gene mutation was significantly associated with response and resistance to HMAs in univariate analyses, with the exception of *IDH1* and *EZH2*, respectively (Table 2). The number of mutations per sample also did not affect response, with patients with three or more mutations having similar response rates to those with fewer than three mutations (Table 1). To further understand the impact of comutated genes on response, we selected cases with the highest number of comutated genes in our cohort (Data Supplement). None of these combinations predicted response or resistance to HMAs (Data Supplement).

The impact of mutations on response was assessed after controlling for clinical variables such as age and IPSS-R scoring system, using logistic regression analyses. No mutation was associated with overall resistance or response to HMAs, even after adjustment for clinical variables (age, IPSS-R, and sex; Data Supplement).

### Recommender System Genomic Biomarkers That Predict Response

To build strong association rules (associations between genes and outcomes [response *v* no response]), we used strict criteria to identify rules with the highest support (how many times the rules appeared in the data set), high confidence (the confidence of the algorithm in the association rule was set at ≥ 95%), and higher lift (a measure that is reflected in the strength of the association: the higher the lift is, the stronger is the association) in the training cohort. On the basis of these criteria, we found eight rules that predicted resistance to HMAs (Table 3). No strong association rules meeting these strict criteria could predict response to HMAs. These genomic biomarkers were present in 53 of 176 patients (30%) with three or more mutations. When the rules were applied to our patient cohort, they predicted resistance to HMAs correctly in 46 of 53 patients (87%) with relevant molecular mutations. Among the 105 patients with lower-risk disease by IPSS-R (low and very low risk groups), 62 (59%) did respond to HMAs. Among nonresponders, 20 patients had three or more mutations/sample, and the biomarkers were present in seven (35%) of their samples. On the contrary, among 262 patients with higher-risk disease per IPSS-R (intermediate, high, very high), 156 (60%) did not respond. Among nonresponders, 60 patients had three or more mutations/sample and the biomarkers were present in 33 (55%) of their samples. The difference in the presence of the biomarkers in lower- versus higher-risk MDS is related to the higher percentage of patients with three or more mutations/sample in the higher-risk (42%) versus the lower-risk (30%) group.

**TABLE 3.**
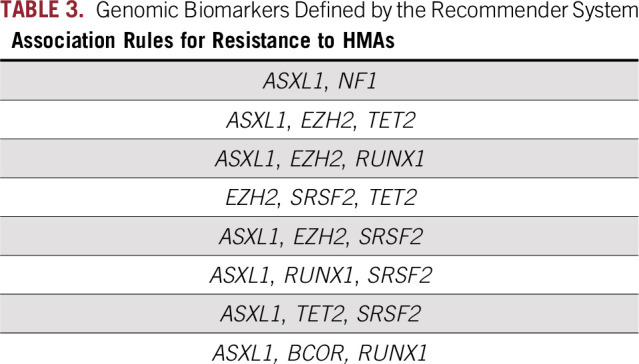
Genomic Biomarkers Defined by the Recommender System

### Association With Overall Survival

**FIG 2.**
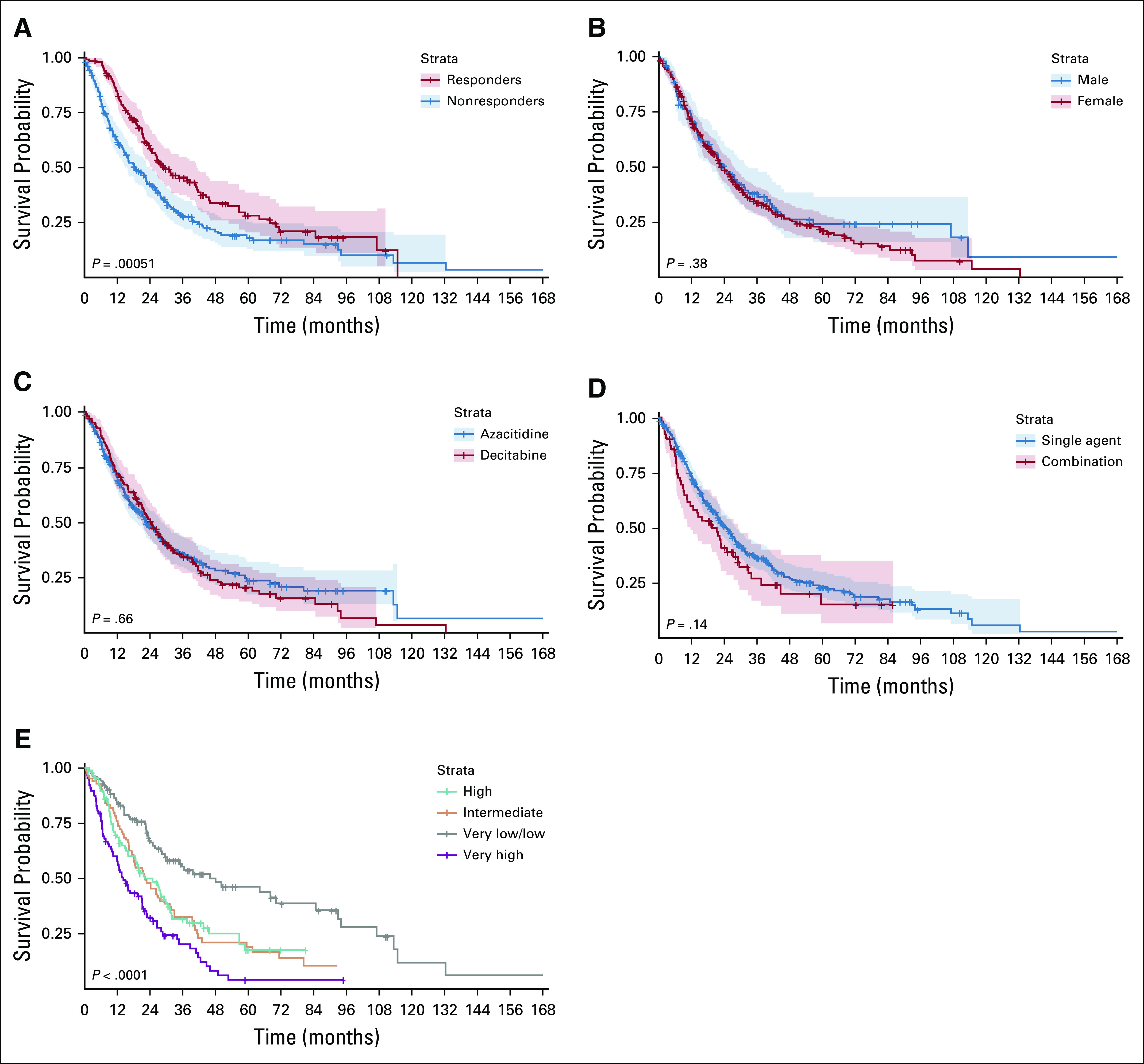
Kaplan-Meier curves for overall survival in our patient cohort with survival data. (A) Survival of responders compared with nonresponders. (B) Survival of male compared with female patients. (C) Survival of patients treated with azacitidine compared with patients treated with decitabine. (D) Survival of patients who received hypomethylating agent as a single agent compared with patients who received it in combination with other drugs. (E) Survival of patients on the basis of International Prognostic Scoring System (IPSS)–revised risk categories.

Survival data were available for 375 patients from the training cohort. With a median follow-up of 30 months (range, 0.62 to 111.7 months), the median OS for the entire group was 19.5 months (95% CI, 9.56 to 34.37 months). The median OS for HMA responders was 29.5 months (95% CI, 25 to 41.3 months) compared with 18.9 months (95% CI, 15 to 24.2 months) for nonresponders (*P* < .001; Fig 2). The median OS was similar for patients treated with AZA (22.9 months [95% CI, 18.9 to 28.4 months]) compared with DAC (24.4 months [95% CI, 21.2 to 29.5 months], *P* = .66; Fig 2). Single-agent HMA versus combinations did not affect survival, with a median OS of 25 months (95% CI, 21.8 to 28.4 months) and 19.7 months (95% CI, 11.8 to 29.2 months), respectively (*P* = .15; Fig 2). The median OS rates per IPSS-R risk categories were 48.1, 22.3, 22.1, and 14.3 months for the low or very low, intermediate, high, and very high subgroups respectively (*P* < .001; Fig 2).

**FIG 3.**
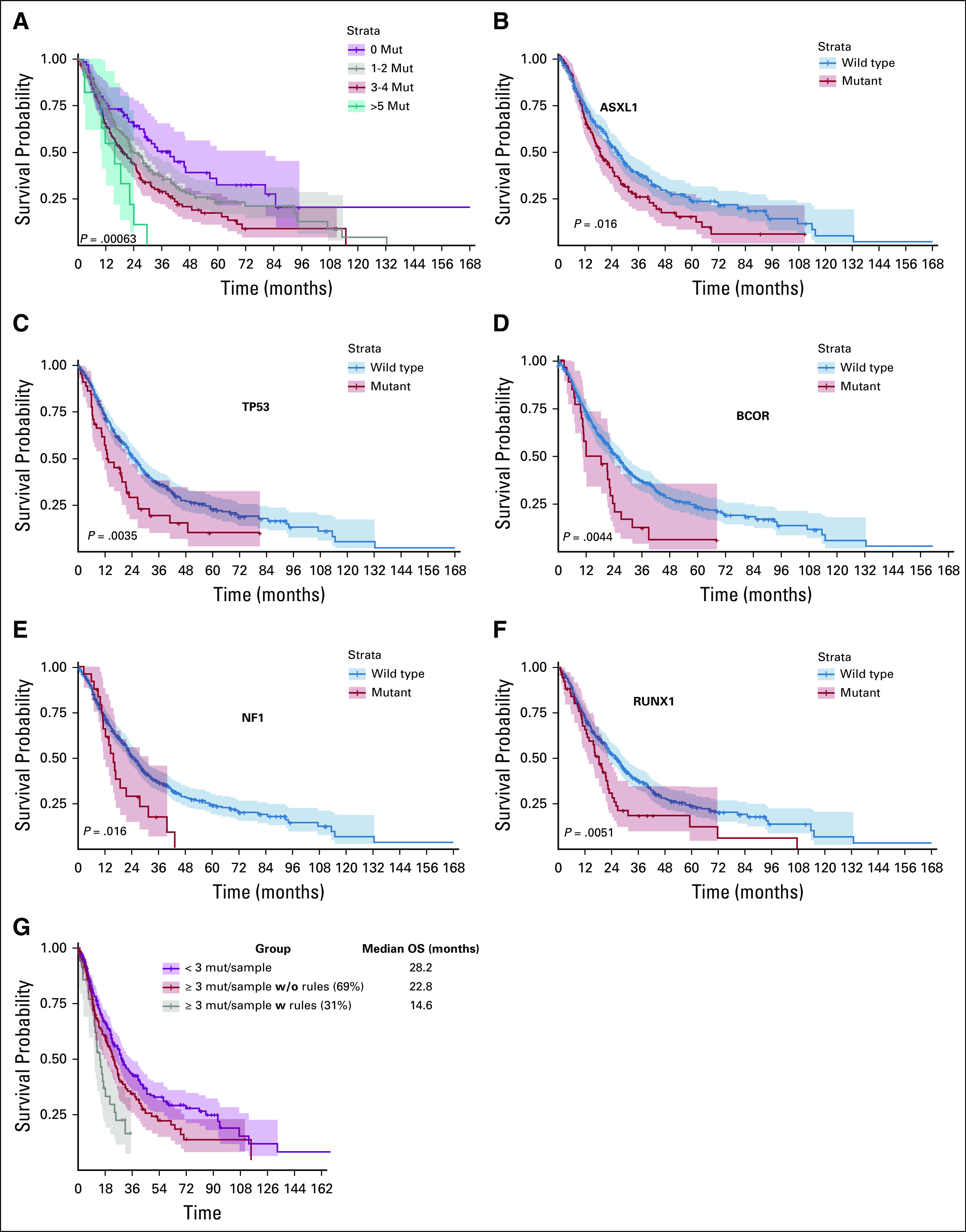
Kaplan-Meier curves for overall survival on the basis of mutations (mut) status. (A) Overall survival (OS) on the basis of the number of mut/sample (regardless of response or resistance to hypomethylating agents). (B) Survival of patients with mutated *ASXL1* compared with wild type. (C) Survival of patients with mutated *TP53* compared with wild type. (D) Survival of patients with mutated *BCOR* compared with wild type. (E) Survival of patients with mutated *NF1* compared with wild type. (F) Survival of patients with mutated *RUNX1* compared with wild type. (G) OS on the basis of the recommender system rules. The graphs compare the OS of patients with three or more mutations and carry one of the proposed rules that were identified in our algorithm (gray line) to patients with three or more mutations without the rules (red line), and patients with fewer than three mutations/sample (purple line).

The median OS for patients with zero mutations/sample was 39.8 months versus 24 months for those with one or two mutations, 19.3 months for those with three to five mutations, and 15.8 months for those with more than five mutations (*P* < .01; Fig 3). Only *ASXL1*, *BCOR*, *DNMT3A*, *RUNX1*, *NF1*, and *TP53* mutations were negatively associated with OS (Fig 3). When applying association rules with an outcome of OS, patients who met at least one of the rules that predicted for resistance had very poor OS compared with patients with three or more mutations/samples who did not meet any of these rules, or patients with fewer than three mutations/sample: 14.6 months versus 22.8 months versus 28.2 months (*P* = .001), respectively (Fig 3).

### Validation of Genomic Biomarkers in Phase II/III Clinical Trial Samples

One hundred three of 113 (91%) in the validation cohort had at least one mutation, the most common being *ASXL1* (n = 31), *TET2* (n = 26), *SRSF2* (n = 23), *TP53* (n = 22), *RUNX1* (n = 21), and *U2AF1* (n=19). The median number of mutations per sample was two (range, zero to seven mutations). Thirty-nine patients (35%) had three or more mutations/sample. Genomic biomarkers of resistance to AZA were present in 14 of 39 samples (35%) with three or more mutations; 13 of 14 of these patients (93%) did not respond to therapy.

## DISCUSSION

Predicting response or resistance to our currently available standard HMA therapy in MDS remains a significant clinical challenge. Identifying patients up front who may not respond to HMAs can potentially improve outcome, decrease unnecessary adverse effects, and save money, especially when current recommendations are for a minimum of 6 months of treatment before deeming it a failure. Although it is tempting to identify an isolated molecular abnormality or a pair of mutations that can predict HMA resistance, this approach does not allow for the complexity and evolution of the genomic landscape in MDS.

In this study, we developed an unbiased framework using a machine learning, recommender system algorithm to identify highly sensitive genomic associations (molecular signatures or genomic biomarkers) that can predict resistance to HMAs with high accuracy. The recommender system algorithm allowed us to identify complex genomic associations that were associated with resistance to HMAs without pregrouping mutations. These associations were validated in an independent cohort in samples from patients enrolled in a randomized phase II/III clinical trial. Although our biomarkers were identified in only 25% of patients, their presence predicted resistance in almost all patients who had these mutations. By definition, a biomarker can be present in a small subset of patients, but when present can predict, with high accuracy and reliability, response or resistance to a therapy. More importantly, our biomarkers also correlated with worse survival, suggesting higher-risk features of disease resistance and progression. Detecting these biomarkers in 29% of patients suggests that other biologic mechanisms (eg, changes in gene expression or epigenetic changes) or clinical characteristics may contribute more to HMA response and failure than does genomics. Indeed, several studies have shown that genomic clonal architecture does not change at the time of response to HMAs in serial samples obtained from patients during therapy. Our findings confirm that genomic associations may lead to different gene expressions and/or epigenetic changes that contribute to the response or resistance and thus, identifying one or two genes that can predict response may not be sufficient to build reliable and predictable models.

Although we included patients who received HMAs in combination with other investigational agents, these combinations did not affect the response or resistance rate or OS; thus, their impact on the output of our recommender system algorithms is negligible. Furthermore, neither IPSS nor IPSS-R predicted response or resistance to HMAs in our study in accordance with prior reports.^1,15^

Prior studies have attempted to use genomic data to predict response or resistance to HMAs. The results among these studies have been controversial. For example, some studies have shown that *TP53* mutations may predict response to HMAs, whereas others did not confirm that finding.^13,17^ In a small study of 84 patients with acute myeloid leukemia (AML) and MDS treated with a 10-day DAC course, a small subset of patients with *TP53* mutations had a higher response rate to DAC compared with *TP53* wild-type patients. Furthermore, the median OS for *TP53* mutated patients who received DAC and underwent an allogeneic stem-cell transplantation was similar to that of patients with wild-type *TP53*^17^. Contrary to this finding, in a study of 71 patients with AML, there was no difference in overall response rate and survival among patients who received 5 days of DAC compared with those who had a 10-day schedule. More importantly, *TP53* status did not affect their response.^18^ Similarly, prior studies have shown that *TET2* mutations with variant allele frequency greater than 10% may predict response to HMAs, especially in patients with wild-type *ASXL1* mutations, but this finding was not validated in our study.^11^ The discrepancy in the results of these studies could be related to sample size, the number of genes tested, and the statistical approach that was used to analyze the data. It is also possible that genomic changes in themselves are not the drivers of response to HMAs but rather, changes in the gene expression and methylation profile that are derived from the combination of these mutations. In a study of whole-genome sequencing, RNA sequencing, and methylation profile of samples from patients with chronic myelomonocytic leukemia, a serial sequencing demonstrates that the response to hypomethylating agents is associated with changes in DNA methylation and gene expression, without any decrease in the mutation allele burden or prevention of new genetic alteration occurrence.^18^

This study includes several areas of innovation. On the clinical side, these genomic biomarkers can be used to tailor therapy. For example, if a patient with MDS with higher-risk disease carries one of these biomarkers, he or she should be encouraged to enroll in a clinical trial with a novel therapy or to proceed with an allogeneic stem-cell transplantation, if eligible, directly, without the use of HMAs, because the response to such therapy is predicted to be low. Although all patients with MDS should be encouraged to enroll in a clinical trial with novel therapies, having biomarkers that accurately predict resistance may ease the conversation with some patients who are hesitant to try newer approaches and prefer Food and Drug Administration–approved therapies.^19^ Alternatively, patients with higher-risk disease and a high blast percentage may consider intensive, AML-type chemotherapy before allogeneic stem-cell transplantation, as opposed to an HMA that is predicted to do little to affect the disease in the absence of other biomarkers that could predict resistance to chemotherapy, such as complex karyotype cytogenetics, and the presence of *TP53* mutations and the absence of targetable mutations such as *IDH1* and *IDH2*. Because the optimal timing for patients with MDS with lower-risk disease can be challenging and because these genomic biomarkers predicted poor outcome even in patients with lower-risk disease. These biomarkers could be used as a justification to proceed with allogeneic stem-cell transplantation early in the disease course, especially if the patient has a lower blast percentage. In addition, identifying, with high accuracy, patients who may or may not respond to therapy can prevent prolonged exposure to ineffective therapy and can lead to lower cost without decreasing value or changing patient outcomes. Translationally, these genomic biomarkers can also be used to model HMA resistance in the laboratory and to study the mechanisms of resistance in cell cultures and animal models. Introducing these biomarkers into normal hematopoietic cells using CRISPR/cas9 may offer an opportunity to model and understand HMA resistance to develop novel drugs to overcome this resistance.

Our study highlights the importance of machine learning algorithms such as the recommender system in translating genomic data into useful clinical tools that can be used by physicians in the clinic.^20^ Nevertheless, some limitations to our approach exist. These limitations include the presence of these genomic abnormalities in only approximately one quarter of patients and the lack of identification of rules that predict response to HMAs. It is possible that the response to HMAs is derived mainly from epigenetic changes and is not dependent on the genomic changes that we studied here.

In summary, our study identified genomic abnormalities that predict response or resistance to HMAs in patients with MDS, and we validated our results in an independent patient cohort treated in a randomized clinical trial. Identification of biomarkers that can provide personalized treatment approaches that can predict response or resistance to cancer therapy remains an important clinical challenge, and future drug development should focus on identifying the subgroup of patients who may benefit the most from any given cancer therapy. Such an approach can aid physicians and their patients in selecting the best available therapy to obtain the best outcome.

## Data Availability

The following represents disclosure information provided by authors of this manuscript. All relationships are considered compensated. Relationships are self-held unless noted. I = Immediate Family Member, Inst = My Institution. Relationships may not relate to the subject matter of this manuscript. For more information about ASCO's conflict of interest policy, please refer to www.asco.org/rwc or ascopubs.org/po/author-center. Open Payments is a public database containing information reported by companies about payments made to US-licensed physicians Open Payments. **Honoraria:** DCI **Consulting or Advisory Role:** Karyopharm Therapeutics, Tolero Pharmaceuticals **Speakers' Bureau:** Novartis, Incyte **Research Funding:** Jazz Pharmaceuticals **Consulting or Advisory Role:** Celgene, Millennium Pharmaceuticals, Syros Pharmaceuticals **Research Funding:** Takeda Pharmaceuticals (Inst), Pfizer (Inst) **Honoraria:** Celgene, Alexion Pharmaceuticals, Abbvie/Genentech, Astex Pharmaceuticals, NeoGenomics Laboratories, Daiichi Sankyo, Forty Seven **Consulting or Advisory Role:** Celgene, Foundation Medicine, NeoGenomics Laboratories, Abbvie/Genentech, Astex Pharmaceuticals, Daiichi Sankyo **Research Funding:** Celgene, Takeda Pharmaceuticals **Travel, Accommodations, Expenses:** Celgene **Consulting or Advisory Role:** Celgene, Glycomimetics, Cascadia Laboratories **Stock and Other Ownership Interests:** Abbvie **Consulting or Advisory Role:** Celgene, Novartis, Daiichi Sankyo, Pfizer, Janssen Pharmaceuticals, Agios, Incyte **Speakers' Bureau:** Novartis, Alexion Pharmaceuticals, Jazz Pharmaceuticals **Travel, Accommodations, Expenses:** Celgene, Incyte, Alexion Pharmaceuticals, Novartis, Jazz Pharmaceuticals, Daiichi Sankyo **Stock and Other Ownership Interests:** Array BioPharma (I) **Honoraria:** Daiichi Sankyo, Summer Road, Stemline Therapeutics **Consulting or Advisory Role:** Pfizer **Consulting or Advisory Role:** Acceleron Pharma, Syros, Otsuka US **Consulting or Advisory Role:** Amphivena, Janssen, Amgen, Astex Pharmaceuticals, Celgene, Genoptix, MedImmune, Novartis, Pfizer, Abbvie, Argenx, Array BioPharma, Bayer AG, Celltrion, Jazz Pharmaceuticals, Orsenix, Genentech/Roche, Sandoz, Actinium Pharmaceuticals, Astellas Pharma, Eisai, Daiichi Sankyo, MEI Pharma, Otsuka, Takeda Pharmaceuticals, Roche, Agios, Trovagene **Research Funding:** Abbvie (Inst), Agios (Inst), Astex Pharmaceuticals (Inst), Celgene (Inst), CTI (Inst), Karyopharm Therapeutics (Inst), MedImmune (Inst), MEI Pharma (Inst), Moffitt (Inst), Novartis (Inst), Onconova Therapeutics (Inst), Pfizer (Inst), Sunesis Pharmaceuticals (Inst), Tensha Therapeutics (Inst), Cellectis (Inst), Cellectis, Janssen (Inst), Amphivena (Inst) **Travel, Accommodations, Expenses:** Amphivena, Astex Pharmaceuticals, Janssen, Pfizer, Array BioPharma, Novartis, Abbvie, Jazz Pharmaceuticals, Celgene, Celltrion, Roche/Genentech, Sandoz, Bayer AG, Clovis Oncology, Amgen, Sunesis Pharmaceuticals, Eisai, Agios **Honoraria:** Celgene, Astex Pharmaceuticals, Acceleron Pharma, Helssin, Abbvie **Consulting or Advisory Role:** Celgene, Astex Pharmaceuticals, Acceleron Pharma, Jazz Pharmaceuticals **Research Funding:** Celgene, Astex Pharmaceuticals **Consulting or Advisory Role:** Agios, Astellas Pharma, Amgen, Celgene, Daiichi Sankyo, Glycomimetics, Immunogen, Incyte, Jazz Pharmaceuticals, Macrogenics, Novartis, Pfizer, Seattle Genetics **Speakers' Bureau:** Agios, Celgene, Incyte, Jazz Pharmaceuticals, Novartis **Research Funding:** Agios (Inst), Amgen (Inst), Daiichi Sankyo (Inst), Glycomimetics (Inst), Immunogen (Inst), Janssen Oncology (Inst), Juno Therapeutics (Inst), Pfizer (Inst), Seattle Genetics (Inst), Takeda Pharmaceuticals (Inst) **Other Relationship:** Glycomimetics, Celgene **Consulting or Advisory Role:** GRAIL **Research Funding:** Celgene, Deerfield Management **Patents, Royalties, Other Intellectual Property:** Patents related to the prediction of risk of cardiovascular disease (Inst) No other potential conflicts of interest were reported.
